# Human papillomavirus vaccination access, coverage and dropout in the Federal District: a time series study, 2013-2023

**DOI:** 10.1590/S2237-96222025v34e20240006.en

**Published:** 2025-04-07

**Authors:** Matheus Santos Melo, Thaís Tâmara Castro e Minuzzi-Souza, Laís de Morais Soares, Allan Dantas dos Santos, Tainá Raiol, Ana Ribeiro

**Affiliations:** 1 Fundação Oswaldo Cruz, Núcleo de Saúde da Mulher, Brasília, DF, Brazil; 2 Universidade de Brasília, Programa de Pós-Graduação em Medicina Tropical, Brasília, DF, Brazil; 3 Ministério da Saúde, Secretaria de Vigilância em Saúde e Ambiente, Departamento de Doenças Transmissíveis, Brasília, DF, Brazil; 4 Universidade Federal de Sergipe, Programa de Pós-Graduação em Enfermagem, Aracaju, SE, Brazil

**Keywords:** Papillomavirus Vaccines, Vaccination Coverage, Time Series Studies, Epidemiology, Public Health Surveillance., Vacunas contra Papilomavirus, Cobertura de Vacunación, Estudios de Series Temporales, Epidemiología, Vigilancia en Salud Pública.

## Abstract

**Objective:**

o analyze the temporal trend of human papillomavirus (HPV) vaccination access, coverage and dropout the Federal District, Brazil, from 2013 to 2023.

**Methods:**

This is a time series study using data made available by the Brazilian National Immunization Program and the Federal District Health Department. Vaccination access, coverage and abandonment indicators were calculated. The segmented linear regression method was applied to analyze temporal trends.

**Results:**

A total of 705,823 doses were administered, 484,386 (68.6%) in females and 221,437 (31.4%) in males. Access, with average annual percentage change (AAPC) of -4.6, (confidence interval [95%CI] -8.3; -3.8), and coverage (AAPC -9.2; 95% CI -12.4; -6.6) decreased during the study period. Dropout increased (AAPC 14.1; 95%CI 11.5; 20.0). Despite the reduction in dropout observed among males (AAPC -7.7; 95%CI -10.5; -5.4), there was an increase in dropout among females (AAPC 8.9; 95%CI 4.8; 13.2).

**Conclusion:**

There was a reduction in HPV vaccination access and coverage and an increase in dropout rates, especially among females. Strategies to reverse these trends must be prioritized.

## Resumen

**Objetivo:**

Analizar la tendencia temporal del acceso, cobertura y abandono de la vacunación contra el virus del papiloma humano (VPH) en el Distrito Federal, Brasil, en el período 2013-2023.

**Métodos:**

Se trata de un estudio de series de tiempo utilizando datos puestos a disposición por el Programa Nacional de Inmunizaciones de Brasil y el Departamento de Salud del Distrito Federal. Se calcularon indicadores de acceso, cobertura y abandono de la vacunación. Se aplicó el método de regresión lineal segmentada para analizar las tendencias temporales.

**Resultados:**

Se administraron 705.823 dosis, 484.386 (68,6%) en el sexo femenino y 221.437 (31,4%) en el sexo masculino. El acceso, con un cambio porcentual anual promedio (CPAP) de -4,6 (intervalo de confianza [IC del 95%] -8,3; -3,8) y la cobertura (CPAP -9,2; IC95% - 12,4; -6,6) disminuyeron durante el periodo de estudio. El abandono aumentó (CPAP 14,1; IC95% 11,5; 20,0). A pesar de la reducción del abandono observada entre el sexo masculino (CPAP -7,7; IC95% -10,5; -5,4), hubo un aumento en el abandono entre el sexo femenino (CPAP 8,9; IC95% 4,8; 13,2).

**Conclusión:**

Hubo una reducción en el acceso y la cobertura contra la vacunación contra el VPH y un aumento en las tasas de abandono, especialmente entre el sexo femenino. Es necesario priorizar las estrategias para revertir estas tendencias.

**Ethical aspects**

This research respected ethical principles, having obtained the following approval data:

This research was conducted exclusively with anonymized and aggregated public domain secondary data. As established by National Health Council Resolution No. 466, dated December 12, 2012, which regulates research involving human beings, the project did not need to be submitted to a research ethics committee, since no data were used that would enable identification of individuals.

## Introduction

Human papillomavirus (HPV) is considered the sexually transmitted infection with the highest incidence globally ^1^. The persistence of this infection is responsible for practically all cases of cervical cancer ^2^. There is increasing evidence of its association with other types of cancer, such as anal, vulvar, oropharyngeal, vaginal and penile cancer ^3^. 

HPV can be classified as high or low risk depending on its oncogenic potential ^1^. Types 6 and 11, which are low risk, have been related to 90% of cases of condylomata acuminata and juvenile recurrent papillomatosis. Types 16 and 18, which are high risk, have been found in 70% of cervical cancers, and are also the most common in cancers associated with HPV in other areas of the body ^1^.

Cervical cancer was considered the fourth most common and fourth leading cause of death among women worldwide, with approximately 604,000 new cases and 342,000 deaths recorded in 2020 ^4^ ^,^ ^5^. In Brazil, this type of cancer represents a public health problem, varying in incidence and mortality between the country’s different Federative Units ^5^. Excluding non-melanoma skin tumors, cervical cancer was the third most common among women, with an age-adjusted incidence rate of 15.3 cases per 100,000 in 2022, and an age-adjusted mortality rate of 4.6 deaths per 100,000 women in 2020 ^5^. In the Federal District, the age-adjusted incidence rate was 9.3 cases per 100,000 women ^6^. The age-adjusted mortality rate was 5 deaths per 100,000 women in 2020 ^6^.

Despite the high rates, HPV infections, and consequently the cancers caused by them, are preventable, with vaccination being the main prevention strategy ^7^. In Brazil, the HPV vaccine (quadrivalent 6, 11, 16 and 18) was included on the national vaccination calendar in 2014, with free distribution by the Brazilian National Health System (Sistema Único de Saúde - SUS), through the National Immunization Program ^8^. HPV vaccination initiatives were implemented in the state of Amazonas and the Federal District with effect from 2013 ^8^. 

The HPV vaccination schedule has undergone several changes since its introduction on the national vaccination calendar. Initially, the recommendation was three doses for adolescents aged 11 to 13 years ^9^. In 2015, this age range was expanded to female adolescents aged 9 to 13 ^9^. In 2016, 14-year-old females were included ^10^. In 2017, coverage was expanded to cover females aged 9 to 14 and males aged 11 to 14 ^10^. After five years of implementation, the schedule was simplified to two doses. From 2022 onwards, the policy was unified to cover both sexes in the 9 to 14 age group ^10^. In 2024, the single dose schedule was adopted for adolescents of both sexes aged 9 to 14 ^9^.

Changes in the HPV vaccination schedule over the years have been strategies adopted by the National Immunization Program to increase adherence, expand the availability of this immunobiological to new target groups and follow the recommendations of international bodies, aligning Brazil with the HPV practices of other countries ^9^ ^,^ ^11^.

Primary Health Care (PHC) teams play a crucial role in promoting vaccination coverage, especially among vulnerable populations. PHC acts as a gateway into the health system, facilitating access to essential services, including vaccination, through strategies such as health education, active tracing of those who missed vaccination, as well as immunization campaigns ^12^. Several obstacles compromise the effectiveness of these actions. Among the most common barriers are the lack of confidence in vaccines, concern about possible adverse effects, the spread of misinformation, lack of awareness and social inequalities, such as those related to sex, race/skin color and place of residence, which worsen disparities in access to vaccination ^13^.

Vaccination hesitancy, fueled by the spread of fake news, intensified during the COVID-19 pandemic, contributing to increased vaccine dropout rates. Factors such as health misinformation and the proliferation of myths related to vaccines have negatively impacted vaccination adherence, resulting in vaccination coverage below target and increasing the vulnerability of specific groups ^14^.

It is crucial to conduct studies that analyze indicators related to vaccination over the years. This is essential for the development of strategies that strengthen vaccination against HPV and reduce the incidence and mortality of cancers caused by it. The Federal District, the setting for this study, has socioeconomic diversity and relevance as the country’s capital, where results can reflect and inform public health policies in other regions. 

The objective of this study was to analyze the temporal trend of HPV vaccination access, coverage and dropout in the Brazilian Federal District between 2013 and 2023.

## Methods

### Design

This was a time series study using public domain secondary data, made available by the Brazilian National Immunization Program, on HPV vaccination of adolescents between 2013 and 2023.

### Background

This study was conducted in the Federal District, which is the Federative Unit that is home to the country’s capital, Brasília ^15^. The Federal District has been organized into 35 administrative regions, which establish the area of government activity to facilitate decentralization and improve the coordination of public services ^16^. 

According to the 2022 national census, the Federal District occupied an area of 5,760.784 km2 and had a population of 2,817,381 inhabitants. Among these, 219,738 were aged 9 to 14, representing 7.8% of the total population ^15^. The Federal District stood out for its high human development index of 0.814 in 2021, classified as very high ^15^. Social inequality was a striking characteristic, varying significantly between administrative regions ^17^. 

Data collection for this study took place through access to public databases, as described in detail in the topic “Data source and measurement”. As they provide information of a secondary nature, duly made available by the responsible institutions, these databases were selected for their relevance and comprehensiveness in relation to indicators of interest in the context of HPV vaccination.

### Participants

The study participants were adolescents vaccinated according to the target populations defined by the Ministry of Health throughout the analyzed period. The cohort began in 2013 for females, the year in which HPV vaccination was introduced for this population in the Federal District. The cohort began in 2017 for males, after this group was included as a target audience by the Ministry of Health.

### Variables, data source and measurement

The variables used in the study were sex, age, dose administered and year of vaccination.

All records of vaccination doses administered in the national territory, including in the Federal District, are input to the e-SUS Primary Care information system and the National Immunization Program Information System. These systems are managed by the Ministry of Health to calculate and monitor indicators related to vaccination ^18^.

Data on vaccination in the period 2014-2022 were obtained from Tabet ^19^. We used the National Calendar Vaccination Panel ^20^ to obtain 2023 data. The 2013 data were taken from the public database made available by the Federal District Health Department, via the Citizen Information Service. All of these information sources provided the data in an aggregated form.

Demographic data were obtained from the Information Technology Department of the Brazilian National Health System, based on information generated by the Brazilian Institute of Geography and Statistics (IBGE) ^15^. The digital cartographic meshes were also obtained from the IBGE and from the Federal District Spatial Data Infrastructure Sector ^15^ ^,^ ^16^.

In this study, the indicators were calculated following the guidelines proposed by the Pan American Health Organization and the World Health Organization for vaccination against HPV in the Region of the Americas ^21^.

In order to measure annual access to vaccination, we calculated the quotient between the number of first doses administered to each cohort in each year of analysis and the target population, whereby the result was multiplied by 100 ^21^. In order to facilitate data presentation and comparison, this access indicator was also called “first dose coverage”. Considering the World Health Organization’s 90.0% vaccination coverage target by 2030, this percentage was used for comparison ^22^.

Annual coverage was calculated by dividing the number of second doses administered in each of the cohorts according to the year under analysis by the target population, multiplying the result by 100 ^21^. 

Annual dropout was calculated as the difference between the sum of the first doses administered to the vaccinated cohorts in each year and the sum of the second doses in the same years ^21^. This difference was divided by the total number of first doses administered to the vaccinated cohorts in each of the years, and the result was multiplied by 100 ^21^.

It is important to highlight that, in order to calculate the indicators for all the cohorts vaccinated, the doses administered in the year prior to the studied period were also considered. This approach aimed to achieve a comprehensive assessment of vaccination coverage over time.

### Study size

The study sample was made up of all people vaccinated against HPV, whose information was recorded on the official information systems of the Ministry of Health and the Federal District Health Department.

### Statistical analysis

The absolute and relative frequencies of the number of vaccines administered according to dose, sex and age were calculated. The indicators were calculated, as indicated in the previous section, for each year of the study period. Due to the aggregated nature of the data collected, it was not possible to assess completeness and consistency, which are attributes of the quality of the data analyzed.

The segmented linear regression method (joinpoint) was applied to analyze temporal trends ^23^. This method enabled analysis of temporal trends and changes in trends over the years, adjusting the data to the smallest possible number of statistically significant inflection points ^23^. The choice of trend change periods was made based on the weighted Bayesian information criterion that determines whether the inclusion of an inflection point significantly improves the model fit ^24^. 

Annual percentage change (APC) was presented for each line segment. Average annual percentage change (AAPC) was calculated to quantify the trend throughout the analyzed time interval, with respective 95% confidence intervals (95%CI) ^24^. Trends were considered to be rising when the APC or AAPC values were positive and significant (when zero was not part of the confidence interval intervals). Trends were considered to be falling when the APC or AAPC values were negative and significant. Trends were considered to be stationary in the remaining situations ^23^ ^,^ ^24^.

R 4.3.2 and Microsoft Excel 365 were used for data storage, descriptive analysis and compiling graphs and tables. QGIS 3.34.4 was used to build the map. Joinpoint Regression SoftwareTM 5.0.2 was used for temporal trend analysis and to create the trend graph.

## Results

During the study period, 705,823 doses of the HPV vaccine were administered to adolescents in the Federal District. Of these, 484,386 (68.6%) were administered to females, namely 270,568 (55.9%) first doses and 213,818 (44.1%) second doses, while 221,437 (31.4%) were administered to males, with 139,388 (62.9%) first doses and 82,049 (37.1%) second doses (Table 1). 

The largest number of doses applied to females was at the age of 9 years, totaling 160,018 doses, which represents 33.0% of the total. This is followed by doses at age 10 (123,670 doses; 25.5%), at age 11 (97,272 doses; 20.1%), at age 12 (66,212 doses; 13.7%), at age 13 (30,214 doses; 6.2%) and at 14 years old (7,000 doses; 1.4%). In males, the age with the highest number of doses was 11 years old, with 82,659 doses or 37.3% of the total. The subsequent ages, in descending order, were 12 years (56,796 doses; 25.7%), 13 years (35,008 doses; 15.8%), 14 years (20,884 doses; 9.4%), 9 years (13,640 doses; 6.2%) and 10 years (12,450 doses; 5.6%) (Table 1).

**Table 1.** Administered doses of human papillomavirus vaccine, by sex, dose and age. Federal District, 2013-2023 (n=705,823)

Females												
Year	First dose						Second dose					
	9 years	10 years	11 years	12 years	13 years	14 years	9 years	10 years	11 years	12 years	13 years	14 years
2013	1,714	14,776	21,939	22,287	6,556	21	926	9,930	20,072	20,544	9,807	23
2014	18,498	19,510	8,606	2,528	1,909	162	10,478	13,838	7,416	1,729	1,260	200
2015	4,069	768	315	124	104	145	170	241	160	113	71	62
2016	12,716	6,003	2,330	907	473	79	3,917	5,639	2,195	921	502	197
2017	10,947	3,284	2,309	1,154	740	264	5,317	6,261	3,314	1,200	642	368
2018	9,752	2,436	1,573	747	365	221	4,342	4,186	2,227	1,335	563	309
2019	9,791	2,740	1,917	699	438	262	4,337	4,257	2,341	1,223	719	378
2020	11,820	3,516	3,354	1,262	685	499	4,387	4,635	3,202	1,893	1,082	793
2021	10,765	2,759	2,398	1,094	539	330	4,333	4,247	2,921	1,667	889	556
2022	10,396	2,389	1,725	830	461	312	3,901	4,123	2,495	1,376	762	519
2023	12,426	3,045	1,678	945	631	531	5,016	5,087	2,785	1,634	1,016	769
Males												
Variables	First dose						Second dose					
	9 years	10 years	11 years	12 years	13 years	14 years	9 years	10 years	11 years	12 years	13 years	14 years
2017	89	107	7,295	11,791	8,401	2,966	15	28	256	2,256	3,656	2,173
2018	108	143	8,906	4,403	2,102	1,573	51	84	3,211	5,364	3,612	2,699
2019	92	124	9,337	2,907	1,567	998	41	46	3,453	3,800	2,420	1,601
2020	69	107	9,906	3,220	1,823	1,317	22	34	3,500	3,811	2,226	1,646
2021	74	42	9,214	2,648	1,096	731	30	36	3,368	3,738	1,849	1,274
2022	1,669	1,480	8,161	2,265	900	560	21	55	2,858	3,466	1,683	961
2023	9,032	6,551	8,080	3,005	1,467	940	1,422	2,763	4,787	4,058	2,139	1,426

**Figure 1.**
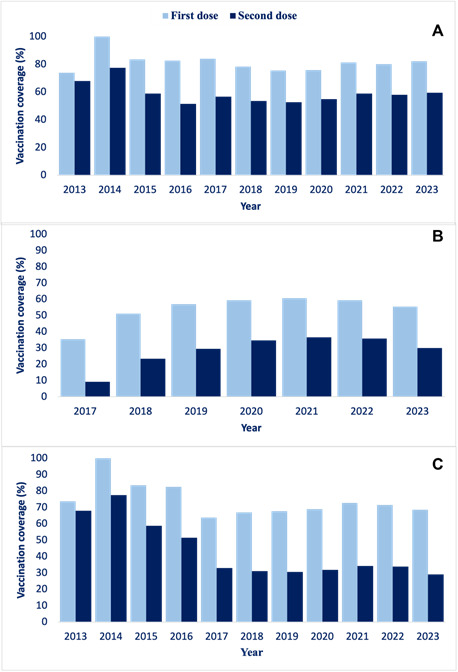
Coverage for females (A), males (B) and total coverage (C) of the first and second administered doses of human papillomavirus vaccine. Federal District, 2013-2023 (n=705,823)

The Federal District did not achieve the complete vaccination schedule coverage target for the population studied in any of the years analyzed. In the case of females, the highest and lowest coverage values for the first dose were 100.0% and 73.8% in 2014 and 2013, respectively. For the second dose, the highest and lowest coverage values were 77.5% and 51.5% in 2014 and 2016, respectively. In the case of males, the lowest and highest percentages of coverage for the first and second doses were 35.4% and 9.2%, in 2017, and 60.7% and 36.7% in 2021 (Figure 1).

Considering the entire study period, the Federal District showed a falling trend in percentage access (AAPC -4.6; 95%CI -8.3; -3.8) and coverage (AAPC -9.2; 95%CI -12.4; -6.6), while the dropout percentage was rising (AAPC 14.1; 95%CI 11.5; 20.0).

**Figure 2.**
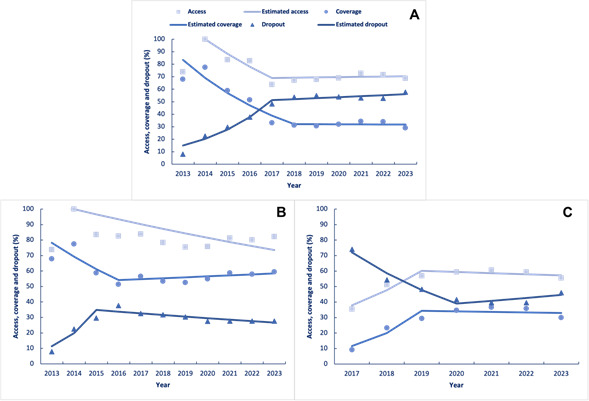
Temporal trend analysis of total (A), female (B) and male (C) human papillomavirus vaccination estimated access, estimated coverage and estimated and observed dropout values. Federal District, 2013-2023

The analyses revealed significant differences between the sexes (Figure 2 and Table 2). Females demonstrated a falling trend for access (AAPC -3.4; 95%CI -7.2; -3.7) and coverage (AAPC -2.9; 95%CI -4.8; -0.7) and a rising trend for dropout (AAPC 8.9; 95%CI 4.8; 13.2). Males showed increased access (AAPC 7.1; 95%CI 3.3; 12.5) and coverage (AAPC 19.0; 95%CI 4.7;43.0) and a fall in dropout (AAPC -7.7; 95%CI -10.5; -5.4).

**Table 2.** Annual percentage change (APC), average annual percentage change (AAPC) and 95% confidence interval (95%CI) of human papillomavirus (HPV) vaccination access, coverage and dropout, by sex. Federal District, Brazil, 2013-2023 (n=705.823)

Variable	Percentage	Segment of the period			Complete period	
		Period	APC (95%CI)	Trend	AAPC (95%CI)	Trend
**Females**						
Access	81.6	2013-2023	-3.4 (-7.2; -3.7)	Falling	-3.4 (-7.2; -3.7)	Falling
Coverage	58.7	2013-2016	-11.5 (-22.3; -4.2)	Falling	-2.9 (-4.8; -0.7)	Falling
		2016-2023	1.1 (-1.6; 10.1)	Stable		
Dropout	28.1	2013-2015	75.6 (32.4; 115.7)	Rising	8.9 (4.8; 13.2)	Rising
		2015-2023	-3.3 (-6.0; -1.1)	Falling		
**Males**						
Access	53.9	2017-2019	26.1 (10.7; 49.3)	Rising	7.1 (3.3; 12.5)	Rising
		2019-2023	-1.2 (-9.2; 2.1)	Stable		
Coverage	28.2	2017-2019	72.2 (16.4; 219.6)	Rising	19.0 (4.7; 43.0)	Rising
		2019-2023	-1.1 (-27.2; 14.7)	Stable		
Dropout	47.7	2017-2020	-18.5 (-27.4; -13.4)	Falling	-7.7 (-10.5; -5.4)	Falling
		2020-2023	4.6 (-2.9; 20.5)	Stable		
**Total**						
Access	72.2	2013-2017	-11.6 (-31.5; -8.5)	Falling	-4.6 (-8.3; -3.8)	Falling
		2017-2023	0.3 (-4.4; 16.9)	Stable		
Coverage	38.9	2013-2018	-17.4 (-30.1; -12.5)	Falling	-9.2 (-12.4; -6.6)	Falling
		2018-2023	-0.2 (-7.9; 22.9)	Stable		
Dropout	46.2	2013-2017	36.0 (23.9; 67.8)	Rising	14.1 (11.5; 20.0)	Rising
		2017-2023	1.5 (-1.4; 4.2)	Stable		

Data were collected on the main chronic noncommunicable diseases of the 572 patients treated. This resulted in 1,149 reported diseases, which were classified into 12 groups, as shown in Table 3.

## Discussion

HPV vaccination access and coverage in the Federal District was below the 90% target established by the World Health Organization, with downward trends in access and coverage and an increase in dropout, especially among females. 

It is important to consider some of the limitations of this study. Use of secondary data could have led to loss of information and classification bias due to possible errors in records. The absence of variables such as race/skin color, education and place of residence limited the detailed analysis of these influences on vaccine access and dropout. The use of intercensal population estimates could have generated inaccuracies in the indicators calculated. Temporal differences in the availability of vaccination could have affected the comparison between regions, introducing information bias. Variation in the start and availability of vaccination may have impacted the pattern of growth or stabilization of vaccination coverage. This may have led to underestimation or overestimation of differences in the trends of the indicators presented between the Federal District and other regions.

The Federal District data were similar to those of other Federative Units. In Northeast Brazil, vaccination coverage for females was 73.9% for the first dose and 54.3% for the second, while for males it was 49.7% and 32.6% ^25^. Despite low coverage, the Federal District showed a promising reality in relation to the global scenario, in which HPV vaccination coverage was 15.0%, well below the target of 90.0% ^22^. With access close to 70.0%, there were positive prospects for expanding coverage and improving vaccination indicators.

The Ministry of Health decision to adopt the single-dose HPV vaccine schedule with effect from 2024, as recommended by the World Health Organization, has facilitated the increase in vaccination coverage in the Federal District ^9^. While this change is promising, it is essential to implement additional strategies in order to achieve the 90.0% target, which had not been achieved as at 2023.

Expanding vaccination coverage faces several challenges, especially when vaccinating adolescents. Obstacles include parental concerns about vaccine safety, lack of trust, social norms, exposure to rumors and myths, miscommunication from service providers, as well as social inequities and access barriers ^26^. PHC plays a fundamental role in this context. In municipalities with greater PHC and Family Health Strategy coverage, in addition to a greater number of community health agents, vaccination coverage targets are more frequently achieved ^12^.

The implementation of combined strategies in PHC contributes to increasing vaccination adherence and increasing vaccination coverage. These strategies include health education, active tracing of individuals who missed vaccination and intensification of vaccination actions. Furthermore, effective communication and public awareness, together with continuous monitoring of immunization indicators, strengthen the positive impact of these actions ^27^. 

Parental difficulty in taking adolescents for vaccination during business hours is a significant obstacle ^28^. In order to overcome this challenge, it is crucial to offer vaccines in alternative locations, such as schools, urgent care services, hospitals and pharmacies ^29^. This strategy requires adequate funding to ensure the provision of preventive care based on health education and evidence ^28^.

Strengthening national programs such as the School Health Program (Programa Saúde na Escola) can improve vaccination coverage in the Federal District ^28^. Vaccination in schools, when integrated with public health campaigns and combined with communication and education strategies, is effective in increasing adolescent adherence ^30^. The growing autonomy of young people can reduce this adherence, especially if there is misinformation. In order to mitigate this risk, it is essential to reformulate educational strategies, providing clear and scientific information that empowers young people to make informed decisions and get involved in health campaigns ^31^ ^,^ ^32^.

Although no significant inflection points were identified during the COVID-19 pandemic, this period negatively impacted vaccination coverage, including HPV vaccination. Restriction measures resulted in a decrease in supply and access to immunization services. The spread of fake news about vaccines has contributed to falling vaccination rates through increased vaccination hesitancy ^14^.

Females had higher coverage rates, but an increase in vaccination dropout was also identified among them. This behavior could be related to the way vaccination was initially structured for this target audience. Vaccination started in schools, favoring achievement of high first dose coverage. In 2014, reported psychogenic reactions undermined confidence in the vaccine, negatively impacting second dose coverage and subsequent indicators. From 2016 onwards, the simplification of the vaccination schedule, from three to two doses, contributed to stabilization of coverage and reduction of dropouts ^33^. In the case of males, the inclusion of the meningococcal serogroup C vaccine on the vaccination schedule in 2017 may have influenced vaccination behavior, increasing adherence to the HPV vaccine. This effect was explained by greater familiarity with vaccination campaigns and the positive perception associated with vaccination against serious diseases such as meningitis ^34^. 

Public policies aimed at expanding vaccination in both sexes, based on the situation found in this study and evidence of the potential to reduce forms of cancer associated with vaccination, are essential and should be strengthened ^35^. These policies should include multimodal educational actions, such as websites, social networks and other interactive tools, to raise awareness among adolescents and their families about the importance of vaccination, reducing hesitancy and concerns about side effects.

In conclusion, HPV vaccination coverage in the Federal District was low, representing a significant public health challenge, especially among male adolescents, who had the poorest indicators. It is crucial to strengthen health surveillance actions and implement educational strategies to increase vaccination adherence.

## Data Availability

The databases used in this research are available at https://www.ebi.ac.uk/biostudies/studies/S-BSST1683.
