# Supplementary material for: Human papillomavirus vaccination access, coverage and dropout in the Federal District: a time series study, 2013-2023
Source: Epidemiol Serv Saude. 2025 Apr 7;34:e20240006. doi: 10.1590/S2237-96222025v34e20240006.en (PMC11978278; doi:10.1590/S2237-96222025v34e20240006.en)
Supplement: Translation

# Translation: Acesso, cobertura e abandono da vacinação contra o papilomavírus humano no Distrito Federal: estudo de série temporal, 2013-2023

**Authors:** Matheus Santos Melo, Thaís Tâmara Castro e Minuzzi-Souza, Laís de Morais Soares, Allan Dantas dos Santos, Tainá Raiol, Ana Ribeiro

**Affiliations:** 1 Fundação Oswaldo Cruz, Núcleo de Saúde da Mulher, Brasília, DF, Brazil; 2 Universidade de Brasília, Programa de Pós-Graduação em Medicina Tropical, Brasília, DF, Brazil; 3 Ministério da Saúde, Secretaria de Vigilância em Saúde e Ambiente, Departamento de Doenças Transmissíveis, Brasília, DF, Brazil; 4 Universidade Federal de Sergipe, Programa de Pós-Graduação em Enfermagem, Aracaju, SE, Brazil

**Keywords:** Vacinas contra Papilomavírus, Cobertura Vacinal, Estudos de Séries Temporais, Epidemiologia, Vigilância em Saúde Pública.

## Abstract

**Objetivo:**

Analisar a tendência temporal de acesso, cobertura e abandono da vacinação contra o papilomavírus humano (human papillomavirus, HPV) no Distrito Federal no período 2013-2023.

**Métodos:**

Trata-se de estudo de série temporal utilizando dados disponibilizados pelo Programa Nacional de Imunizações do Brasil e pela Secretaria de Saúde do Distrito Federal. Os indicadores de acesso, cobertura e abandono vacinal foram calculados. Aplicou-se o método de regressão linear segmentada para a análise das tendências temporais.

**Resultados:**

Foram administradas 705.823 doses, sendo 484.386 (68,6%) no sexo feminino e 221.437 (31,4%) no sexo masculino. O acesso, com variação percentual anual média (VPAM) de -4,6,(intervalo de confiança [IC95%] -8,3; -3,8), e a cobertura (VPAM -9,2; IC95% -12,4; -6,6) reduziram no período de estudo. O abandono cresceu (VPAM 14,1; IC95% 11,5; 20,0). Apesar da redução do abandono observada no sexo masculino (VPAM -7,7; IC95% -10,5; -5,4), houve crescimento do abandono no sexo feminino (VPAM 8,9; IC95% 4,8; 13,2). **Conclusão:** Observou-se redução no acesso e na cobertura vacinal contra o HPV e aumento no abandono, especialmente no sexo feminino. Estratégias para reversão dessas tendências devem ser priorizadas.

## Introdução

A infecção pelo papilomavírus humano (human papillomavirus, HPV) é considerada a doença sexualmente transmissível com maior incidência em todo o mundo ^1^. A persistência dessa infecção é responsável por praticamente todos os casos de câncer do colo do útero ^2^. Há evidências crescentes de sua associação com outros tipos de câncer, tais como anal, vulvar, orofaríngeo, vaginal e peniano ^3^. 

O HPV pode ser classificado em alto ou baixo risco conforme seu potencial oncogênico ^1^. Os tipos 6 e 11, de baixo risco, relacionaram-se a 90% dos casos de condilomas acuminados e papilomatose recorrente juvenil. Os tipos 16 e 18, de alto risco, encontravam-se em 70% dos cânceres de colo do útero, sendo também os mais incidentes em cânceres associados ao HPV em outras áreas do corpo ^1^.

O câncer do colo do útero foi considerado o quarto mais comum e a quarta principal causa de morte entre mulheres em todo o mundo, com cerca de 604 mil novos casos e 342 mil mortes registradas em 2020 ^4^ ^,^ ^5^. No Brasil, esse tipo de câncer representa um problema de saúde pública, variando em incidência e mortalidade entre as diferentes unidades da Federação ^5^. Excluindo tumores de pele não melanoma, ele foi o terceiro mais comum entre as mulheres, com uma taxa de incidência ajustada por idade de 15,3 casos por 100 mil em 2022, e a taxa de mortalidade ajustada por idade de 4,6 óbitos por 100 mil mulheres em 2020 ^5^. No Distrito Federal, a taxa de incidência ajustada por idade foi de 9,3 casos por 100 mil mulheres ^6^. A taxa de mortalidade ajustada por idade foi de 5 óbitos por 100 mil mulheres em 2020 ^6^.

Apesar das altas taxas, as infecções por HPV, e consequentemente os cânceres por ela causados, são preveníveis, com vacinação sendo a principal estratégia de prevenção ^7^. No Brasil, a vacina contra o HPV (quadrivalente 6, 11, 16 e 18) foi incluída ao calendário nacional de vacinação em 2014, com a distribuição gratuita pelo Sistema Único de Saúde, através do Programa Nacional de Imunizações ^8^. Iniciativas de vacinação foram implementadas no Amazonas e no Distrito Federal a partir de 2013 ^8^. 

O esquema vacinal contra o HPV passou por várias mudanças desde sua introdução no calendário nacional de vacinação. Inicialmente, a recomendação era de três doses para adolescentes de 11 a 13 anos ^9^. Em 2015, essa faixa etária foi ampliada para adolescentes de 9 a 13 anos do sexo feminino ^9^. Em 2016, incluiu-se o sexo feminino de 14 anos ^10^. Em 2017, expandiu-se a cobertura para abranger indivíduos do sexo feminino de 9 a 14 anos e do sexo masculino de 11 a 14 anos ^10^. Após cinco anos de implementação, o esquema foi simplificado para duas doses. A partir de 2022, a política foi unificada para contemplar ambos os sexos na faixa etária de 9 a 14 anos ^10^. Em 2024, foi adotado o esquema de dose única para adolescentes de 9 a 14 anos de ambos os sexos ^9^.

As alterações no esquema de vacinação contra o HPV ao longo dos anos foram estratégias adotadas pelo Programa Nacional de Imunizações para aumentar a adesão, expandir a oferta do imunobiológico a novos grupos-alvo e seguir as recomendações de órgãos internacionais, alinhando o Brasil às práticas de outros países ^9^ ^,^ ^11^.

As equipes de Atenção Primária à Saúde (APS) desempenham papel crucial na promoção da cobertura vacinal, especialmente entre populações vulneráveis. A APS atua como porta de entrada para o sistema de saúde, facilitando o acesso a serviços essenciais, incluindo a vacinação, por meio de estratégias como educação em saúde, busca ativa de faltosos e campanhas de imunização ^12^. Diversos obstáculos comprometem a eficácia dessas ações. Entre as barreiras mais comuns, estão a falta de confiança nas vacinas, a preocupação com possíveis efeitos adversos, a disseminação de desinformação, a falta de conscientização e as desigualdades sociais, como as relacionadas ao sexo, raça/cor da pele e local de moradia, que agravam as disparidades no acesso à vacinação ^13^.

A hesitação vacinal, alimentada pela disseminação de notícias falsas, intensificou-se durante a pandemia de covid-19, contribuindo para o aumento dos índices de abandono vacinal. Fatores como a desinformação em saúde e a proliferação de mitos relacionados às vacinas têm impactado negativamente a adesão à vacinação, resultando em coberturas vacinais abaixo da meta e aumentando a vulnerabilidade de grupos específicos ^14^.

É crucial conduzir estudos que analisem os indicadores relacionados à vacinação ao longo dos anos. Isso é fundamental para o desenvolvimento de estratégias que fortaleçam a vacinação contra o HPV e reduzam a incidência e mortalidade dos cânceres por ele causados. O Distrito Federal, cenário deste estudo, possui diversidade socioeconômica e relevância como capital do país, onde os resultados podem refletir e informar políticas de saúde pública em outras regiões. 

Este estudo teve como objetivo analisar a tendência temporal de acesso, cobertura e abandono da vacinação contra o HPV no Distrito Federal no período 2013-2023.

## Métodos

### Delineamento 

Tratou-se de estudo de série temporal utilizando dados secundários de domínio público, disponibilizados pelo Programa Nacional de Imunizações do Brasil, sobre a vacinação contra o HPV de adolescentes no período 2013-2023.

### Contexto

Este estudo foi conduzido no Distrito Federal, Unidade da Federação que sedia a capital do país, Brasília ^15^. Essa unidade foi organizada em 35 regiões administrativas, que estabelecem a área de atuação governamental para facilitar a descentralização e melhorar a coordenação dos serviços públicos ^16^. 

De acordo com o censo nacional de 2022, o Distrito Federal ocupava a área de 5.760,784 km2 e tinha população de 2.817.381 habitantes. Entre estes, 219.738 tinham 9 a 14 anos, representando 7,8% do total populacional da unidade ^15^. O Distrito Federal destacou-se por seu elevado índice de desenvolvimento humano de 0,814 em 2021, classificado como muito alto ^15^. A desigualdade social foi característica marcante, variando significativamente entre as regiões administrativas ^17^. 

A coleta de dados deste estudo foi realizada por meio do acesso a bancos de dados públicos, conforme descrito detalhadamente no tópico “Fonte de dados e mensuração”. Por fornecerem informações de natureza secundária, devidamente disponibilizadas pelas instituições responsáveis, esses bancos de dados foram selecionados por sua relevância e abrangência em relação aos indicadores de interesse no contexto da vacinação contra o HPV.

### Participantes

Os participantes do estudo foram os adolescentes vacinados conforme as populações-alvo definidas pelo Ministério da Saúde ao longo do período analisado. A coorte iniciou-se em 2013 para o sexo feminino, ano em que a vacinação contra o HPV foi introduzida para esse público no Distrito Federal. A coorte teve início em 2017 para o sexo masculino, após a inclusão desse grupo como público-alvo pelo Ministério da Saúde.

### Variáveis, fonte de dados e mensuração

As variáveis utilizadas no estudo foram sexo, idade, dose aplicada e ano da vacinação.

Todos os registros de doses vacinais realizadas no território nacional, incluindo no Distrito Federal, foram inseridos nos sistemas de informações e-SUS Atenção Primária e Sistema de Informação do Programa Nacional de Imunizações. Esses sistemas são geridos pelo Ministério da Saúde para cálculo e monitoramento dos indicadores relacionados à vacinação ^18^.

Os dados sobre a vacinação no período 2014-2022 foram obtidos do Tabnet ^19^. Para os dados de 2023, utilizou-se o Painel de Vacinação do Calendário Nacional ^20^. Os dados de 2013 foram encontrados no banco de dados público disponibilizado pela Secretaria de Saúde do Distrito Federal, via Serviço de Informação ao Cidadão. Todas essas fontes de informação forneceram os dados de forma agregada.

Os dados demográficos foram obtidos do Departamento de Informática do Sistema Único de Saúde, a partir de informações geradas pelo Instituto Brasileiro de Geografia e Estatística (15). As malhas cartográficas digitais foram obtidas desse mesmo instituto e do setor de Infraestrutura de Dados Espaciais do Distrito Federal ^15^ ^,^ ^16^.

Neste estudo, os indicadores foram calculados seguindo as diretrizes propostas pela Organização Pan-Americana da Saúde e pela Organização Mundial da Saúde para a vacinação contra o HPV na Região das Américas ^21^.

Para mensurar o acesso anual à vacinação, calculou-se o quociente entre o número de primeiras doses administradas a cada coorte em cada ano de análise e a população-alvo, resultado este multiplicado por 100 ^21^. A fim de facilitar a apresentação e comparação dos dados, esse indicador de acesso foi também denominado como “cobertura de primeira dose”. Considerando a meta de cobertura vacinal da Organização Mundial da Saúde para 2030 de 90,0%, esse valor foi utilizado para comparação ^22^.

A cobertura anual foi calculada pela divisão entre o número de segundas doses administradas em cada uma das coortes, segundo ano sob análise, e a população-alvo, multiplicando-se o resultado por 100 ^21^. 

O abandono anual foi calculado pela diferença do somatório das primeiras doses administradas para as coortes de vacinados em cada um dos anos e do somatório das segundas doses nos mesmos anos ^21^. Essa diferença foi dividida pelo total de primeiras doses administradas para as coortes de vacinados em cada um dos anos, e o resultado, multiplicado por 100 ^21^.

É importante destacar que, para o cálculo dos indicadores de todas as coortes de vacinados, também foram consideradas as doses administradas no ano anterior ao período estudado. Essa abordagem visou a uma avaliação abrangente da cobertura vacinal ao longo do tempo.

### Tamanho do estudo

A amostra do estudo foi composta por todas as pessoas vacinadas contra o HPV, cujas informações foram registradas nos sistemas oficiais de informação do Ministério da Saúde e da Secretaria de Saúde do Distrito Federal.

### Análise estatística

Calcularam-se as frequências absolutas e relativas do número de vacinas administradas segundo dose, sexo e idade. Procedeu-se ao cálculo dos indicadores, conforme indicado na seção anterior, para cada ano do período de estudo. Devido à natureza agregada dos dados coletados, não foi possível realizar a avaliação da completude e da consistência, que são atributos da qualidade dos dados analisados.

O método de regressão linear segmentada (joinpoint) foi aplicado para a análise das tendências temporais ^23^. Esse método permitiu a análise das tendências temporais e das mudanças nas tendências ao longo dos anos, ajustando os dados ao menor número de pontos de inflexão possíveis estatisticamente significativos ^23^. A escolha dos períodos de mudança de tendência foi feita com base no critério de informação bayesiano ponderado que determina se a inclusão de um ponto de inflexão melhora significativamente o ajuste do modelo ^24^. 

A variação percentual anual (VPA) foi apresentada para cada segmento de linha. A variação percentual anual média (VPAM) foi calculada para quantificar a tendência durante todo o intervalo de tempo analisado, com respectivos intervalos de confiança de 95% (IC95%) ^24^. Consideraram-se tendências crescentes quando os valores de VPA ou VPAM foram positivos e significativos (intervalos de confiança não incluem o valor zero). Consideraram-se tendências decrescentes quando os valores de VPA ou VPAM foram negativos e significativos. Tendências estacionárias foram consideradas nas demais situações ^23^ ^,^ ^24^.

O R 4.3.2 e o Microsoft Excel 365 foram utilizados para armazenamento de dados, análise descritiva e construção de gráficos e tabelas. Utilizou-se o QGIS 3.34.4 para construção do mapa. O Joinpoint Regression SoftwareTM 5.0.2 foi utilizado para análise de tendência temporal e elaboração do gráfico de tendência.

## Resultados

No período de estudo, foram administradas 705.823 doses da vacina contra o HPV em adolescentes no Distrito Federal. Destas, 484.386 (68,6%) foram administradas no sexo feminino, sendo 270.568 (55,9%) primeiras doses e 213.818 (44,1%) de segundas doses. Ao passo que, 221.437 (31,4%) foram administradas no sexo masculino, sendo 139.388 (62,9%) primeiras doses e 82.049 (37,1%) segundas doses (Tabela 1). 

A maior quantidade de doses aplicadas no sexo feminino foi para a idade de 9 anos, totalizando 160.018 doses, o que representa 33,0% do total. Isso é seguido por doses aos 10 anos (123.670 doses; 25,5%), aos 11 anos (97.272 doses; 20,1%), aos 12 anos (66.212 doses; 13,7%), aos 13 anos (30.214 doses; 6,2%) e aos 14 anos (7.000 doses; 1,4%). No sexo masculino, a idade com maior número de doses foi a de 11 anos, com 82.659 doses ou 37,3% do total. As idades subsequentes, em ordem decrescente, são 12 anos (56.796 doses; 25,7%), 13 anos (35.008 doses; 15,8%), 14 anos (20.884 doses; 9,4%), 9 anos (13.640 doses; 6,2%) e 10 anos (12.450 doses; 5,6%) (Tabela 1).

**Tabela 1:** Doses aplicadas da vacina contra o papilomavírus humano segundo sexo, dose e idade. Distrito Federal, 2013-2023 (n=705.823)

Sexo feminino												
Variáveis	Primeira dose						Segunda dose					
	9 anos	10 anos	11 anos	12 anos	13 anos	14 anos	9 anos	10 anos	11 anos	12 anos	13 anos	14 anos
2013	1,714	14,776	21,939	22,287	6,556	21	926	9,930	20,072	20,544	9,807	23
2014	18,498	19,510	8,606	2,528	1,909	162	10,478	13,838	7,416	1,729	1,260	200
2015	4,069	768	315	124	104	145	170	241	160	113	71	62
2016	12,716	6,003	2,330	907	473	79	3,917	5,639	2,195	921	502	197
2017	10,947	3,284	2,309	1,154	740	264	5,317	6,261	3,314	1,200	642	368
2018	9,752	2,436	1,573	747	365	221	4,342	4,186	2,227	1,335	563	309
2019	9,791	2,740	1,917	699	438	262	4,337	4,257	2,341	1,223	719	378
2020	11,820	3,516	3,354	1,262	685	499	4,387	4,635	3,202	1,893	1,082	793
2021	10,765	2,759	2,398	1,094	539	330	4,333	4,247	2,921	1,667	889	556
2022	10,396	2,389	1,725	830	461	312	3,901	4,123	2,495	1,376	762	519
2023	12,426	3,045	1,678	945	631	531	5,016	5,087	2,785	1,634	1,016	769
Sexo masculino	Primeira dose						Segunda dose					
Variáveis	9 anos	10 anos	11 anos	12 anos	13 anos	14 anos	9 anos	10 anos	11 anos	12 anos	13 anos	14 anos
2017	89	107	7.295	11.791	8.401	2.966	15	28	256	2.256	3.656	2.173
2018	108	143	8.906	4.403	2.102	1.573	51	84	3.211	5.364	3.612	2.699
2019	92	124	9.337	2.907	1.567	998	41	46	3.453	3.800	2.420	1.601
2020	69	107	9.906	3.220	1823	1.317	22	34	3.500	3.811	2.226	1.646
2021	74	42	9.214	2.648	1.096	731	30	36	3.368	3.738	1.849	1.274
2022	1.669	1.480	8.161	2.265	900	560	21	55	2.858	3.466	1.683	961
2023	9.032	6.551	8.080	3.005	1.467	940	1.422	2.763	4.787	4.058	2.139	1.426

O Distrito Federal não alcançou a meta de cobertura do esquema vacinal completo para população estudada em nenhum dos anos analisados. Para o público feminino, os maiores e menores valores de cobertura de primeira dose foram 100,0% e 73,8% observados em 2014 e 2013. Para a segunda dose, os maiores e menores valores de cobertura foram 77,5% e 51,5% observados em 2014 e 2016. Para o público masculino, os percentuais mais baixos e mais altos de cobertura para a primeira e segunda doses foram 35,4% e 9,2%, em 2017, e de 60,7% e 36,7% em 2021 (Figura 1).

**Figura 1.** Coberturas no sexo feminino (A), masculino (B) e total (C) da primeira dose e da segunda dose da vacina contra o papilomavírus humano administradas. Distrito Federal, 2013-2023 (n=705.823)

Considerando todo o período de estudo, o Distrito Federal apresentou tendência decrescente no percentual de acesso (VPAM -4,6; IC95% -8,3; -3,8) e de cobertura (VPAM -9,2; IC95% -12,4; -6,6), enquanto o percentual de abandono foi crescente (VPAM 14,1; IC95% 11,5; 20,0). 

As análises revelaram diferenças significativas entre os sexos (Figura 2 e Tabela 2). O sexo feminino demonstrou tendência decrescente para o acesso (VPAM -3,4; IC95% -7,2; -3,7) e para a cobertura (VPAM -2,9; IC95% -4,8; -0,7) e crescente para o abandono (VPAM 8,9; IC95% 4,8; 13,2). O sexo masculino apresentou crescimento no acesso (VPAM 7,1; IC95% 3,3; 12,5) e na cobertura (VPAM 19,0; IC95% 4,7; 43,0) e redução no abandono (VPAM -7,7; IC95% -10,5; -5,4).

**Figura 2.** Valores observados e estimados em análise de tendência temporal do acesso, cobertura e abandono da vacinação contra o papilomavírus humano total (A), para o sexo feminino (B) e masculino (C). Distrito Federal

**Tabela 2:** Variação percentual anual (VPA), variação percentual anual média (VPAM) e intervalo de confiança 95% (IC95%) do acesso, da cobertura e do abandono da vacinação contra o papilomavírus humano (HPV) por sexo. Distrito Federal, 2013-2023 (n=705.823)

Variável	Percentual	Segmento do período			Período completo	
		Período	VPA (IC95%)	Tendência	VPAM (IC95%)	Tendência
Sexo feminino						
Acesso	81,6	2013-2023	-3,4 (-7,2; -3,7)	Decrescente	-3,4 (-7,2; -3,7)	Decrescente
Cobertura	58,7	2013-2016	-11,5 (-22,3; -4,2)	Decrescente	-2,9 (-4,8; -0,7)	Decrescente
		2016-2023	1,1 (-1,6; 10,1)	Estável		
Abandono	28,1	2013-2015	75,6 (32,4; 115,7)	Crescente	8,9 (4,8; 13,2)	Crescente
		2015-2023	-3,3 (-6,0; -1,1)	Decrescente		
Sexo masculino						
Acesso	53,9	2017-2019	26,1 (10,7; 49,3)	Crescente	7,1 (3,3; 12,5)	Crescente
		2019-2023	-1,2 (-9,2; 2,1)	Estável		
Cobertura	28,2	2017-2019	72,2 (16,4; 219,6)	Crescente	19,0 (4,7; 43,0)	Crescente
		2019-2023	-1,1 (-27,2; 14,7)	Estável		
Abandono	47,7	2017-2020	-18,5 (-27,4; -13,4)	Decrescente	-7,7 (-10,5; -5,4)	Decrescente
		2020-2023	4,6 (-2,9; 20,5)	Estável		
**Total**						
Acesso	72,2	2013-2017	-11,6 (-31,5; -8,5)	Decrescente	-4,6 (-8,3; -3,8)	Decrescente
		2017-2023	0,3 (-4,4; 16,9)	Estável		
Cobertura	38,9	2013-2018	-17,4 (-30,1; -12,5)	Decrescente	-9,2 (-12,4; -6,6)	Decrescente
		2018-2023	-0,2 (-7,9; 22,9)	Estável		
Abandono	46,2	2013-2017	36,0 (23,9; 67,8)	Crescente	14,1 (11,5; 20,0)	Crescente
		2017-2023	1,5 (-1,4; 4,2)	Estável		

## Discussão

O Distrito Federal apresentou acesso e cobertura vacinal contra o HPV abaixo da meta de 90% estabelecida pela Organização Mundial da Saúde, com tendências de queda do acesso e da cobertura e aumento do abandono, especialmente no sexo feminino. 

É importante considerar algumas limitações deste estudo. A utilização de dados secundários pôde ter levado à perda de informações e ao viés de classificação devido a possíveis erros nos registros. A ausência de variáveis como raça/cor da pele, escolaridade e local de residência limitou a análise detalhada dessas influências no acesso e abandono vacinal. O uso de estimativas populacionais intercensitárias pôde ter gerado imprecisões nos indicadores calculados. Diferenças temporais na oferta da vacinação puderam ter afetado na comparação entre as regiões, introduzindo o viés de informação. A variação no início e na disponibilidade da vacinação pode ter impactado o padrão de crescimento ou estabilização da cobertura vacinal. Isso levou à subestimação ou à superestimação das diferenças nas tendências dos indicadores apresentados entre o Distrito Federal e outras regiões.

Os dados do Distrito Federal foram semelhantes aos de outras unidades da Federação. No Nordeste, a cobertura vacinal para o sexo feminino foi 73,9% na primeira dose e 54,3% na segunda, enquanto, para o masculino, foi 49,7% e 32,6% ^25^. Apesar das baixas coberturas, o Distrito Federal apresentou realidade promissora em relação ao cenário global, em que a cobertura vacinal contra o HPV foi 15,0%, muito abaixo da meta de 90,0% ^22^. Com acesso próximo a 70,0%, houve perspectivas positivas para a ampliação da cobertura e melhoria dos indicadores de vacinação.

A decisão do Ministério da Saúde de adotar o esquema de dose única da vacina contra o HPV a partir de 2024, conforme recomendado pela Organização Mundial da Saúde, facilitou o aumento da cobertura vacinal no Distrito Federal ^9^. Apesar de essa mudança ser promissora, é essencial implementar estratégias adicionais para atingir a meta de 90,0%, que não foi alcançada até 2023.

A ampliação das coberturas vacinais enfrenta vários desafios, especialmente na vacinação de adolescentes. Entre os obstáculos estão preocupações dos pais sobre a segurança das vacinas, falta de confiança, normas sociais, exposição a rumores e mitos, falhas na comunicação por parte dos prestadores de serviços, além de iniquidades sociais e barreiras de acesso ^26^. A APS desempenha papel fundamental nesse contexto. Em municípios com maior cobertura da APS e da Estratégia Saúde da Família, além de maior número de agentes comunitários, as metas de cobertura vacinal são mais frequentemente alcançadas ^12^.

A implementação de estratégias combinadas na APS contribui para aumentar a adesão à vacinação e elevar a cobertura vacinal. Essas estratégias incluem educação em saúde, busca ativa de indivíduos faltosos e intensificação das ações de vacinação. Além disso, a comunicação eficaz e a sensibilização da população, junto ao monitoramento contínuo dos indicadores de imunização, reforçam o impacto positivo dessas ações ^27^. 

A dificuldade dos pais em levar os adolescentes para a vacinação durante o horário comercial é um obstáculo significativo ^28^. Para superar esse desafio, é crucial oferecer vacinas em locais alternativos, como escolas, unidades de urgência, hospitais e farmácias ^29^. Essa estratégia requer financiamento adequado para garantir a prestação de cuidados preventivos baseados em educação em saúde e evidências ^28^.

O fortalecimento de programas nacionais como o Programa Saúde na Escola pode melhorar as coberturas vacinais no Distrito Federal ^28^. A vacinação em escolas, quando integrada a campanhas de saúde pública e combinada com estratégias de comunicação e educação, é eficaz para aumentar a adesão dos adolescentes ^30^. A crescente autonomia dos jovens pode diminuir essa adesão, especialmente se houver desinformação. Para mitigar esse risco, é essencial reformular as estratégias educativas, fornecendo informações claras e científicas que capacitem os jovens a tomarem decisões informadas e a se envolverem em campanhas de saúde ^31^ ^,^ ^32^.

Embora não tenham sido identificados pontos de inflexão significativos durante a pandemia de covid-19, esse período impactou negativamente as coberturas vacinais, incluindo a vacinação contra o HPV. As medidas restritivas resultaram na diminuição da oferta e do acesso aos serviços de imunização. A disseminação de notícias falsas sobre as vacinas contribuiu para a queda nas taxas de vacinação através do aumento da hesitação vacinal ^14^.

O sexo feminino apresentou maiores taxas de cobertura, mas também foi identificado o aumento no abandono vacinal. Esse comportamento pode estar relacionado à forma como a vacinação foi estruturada inicialmente para esse público-alvo. A oferta começou nas escolas, favorecendo o alcance das altas coberturas de primeira dose. Em 2014, reações psicogênicas relatadas comprometeram a confiança na vacina, impactando negativamente a cobertura da segunda dose e os indicadores subsequentes. A partir de 2016, a simplificação do esquema vacinal, de três para duas doses, contribuiu para a estabilização das coberturas e a redução do abandono ^33^. No caso do sexo masculino, a inclusão da vacina meningocócica C no calendário vacinal em 2017 pode ter influenciado o comportamento vacinal, aumentando a adesão à vacina contra o HPV. Esse efeito foi explicado pela maior familiaridade com as campanhas de vacinação e pela percepção positiva associada à vacinação contra doenças graves como a meningite ^34^. 

Políticas públicas direcionadas à ampliação da vacinação em ambos os sexos, com base na situação encontrada neste estudo e em evidências do potencial de redução dos cânceres associados à vacinação, são essenciais e devem ser fortalecidas ^35^. Essas políticas devem incluir ações educativas multimodais, como websites, redes sociais e outras ferramentas interativas, para conscientizar adolescentes e seus familiares sobre a importância da vacinação, reduzindo a hesitação e preocupações sobre efeitos secundários.

Conclui-se que as coberturas vacinais contra o HPV no Distrito Federal foram baixas, representando um desafio significativo de saúde pública, especialmente entre os adolescentes do sexo masculino, que exibiram os piores indicadores. É crucial fortalecer as ações de vigilância em saúde e implementar estratégias educativas para aumentar a adesão à vacinação.

## Data Availability

Os bancos de dados utilizados na pesquisa estão disponíveis em https://www.ebi.ac.uk/biostudies/studies/S-BSST1683.
